# Application of Plant Viruses as a Biotemplate for Nanomaterial Fabrication

**DOI:** 10.3390/molecules23092311

**Published:** 2018-09-11

**Authors:** Yu Zhang, Yixin Dong, Jinhua Zhou, Xun Li, Fei Wang

**Affiliations:** 1 College of Chemical Engineering, Jiangsu Provincial Key Lab for the Chemistry and Utilization of Agro-Forest Biomass, Nanjing Forestry University, Nanjing 210037, China; kaydong417@163.com (Y.D.); 13770559414@163.com (J.Z.); xunlee@njfu.edu.cn (X.L.); hgwf@njfu.edu.cn (F.W.); 2 Jiangsu Co-Innovation Center of Efficient Processing and Utilization of Forest Resources, Nanjing Forestry University, Nanjing 210037, China

**Keywords:** nanotechnology, plant virus, biotemplate, plant virus nanoparticles, nanomaterials

## Abstract

Viruses are widely used to fabricate nanomaterials in the field of nanotechnology. Plant viruses are of great interest to the nanotechnology field because of their symmetry, polyvalency, homogeneous size distribution, and ability to self-assemble. This homogeneity can be used to obtain the high uniformity of the templated material and its related properties. In this paper, the variety of nanomaterials generated in rod-like and spherical plant viruses is highlighted for the cowpea chlorotic mottle virus (CCMV), cowpea mosaic virus (CPMV), brome mosaic virus (BMV), and tobacco mosaic virus (TMV). Their recent studies on developing nanomaterials in a wide range of applications from biomedicine and catalysts to biosensors are reviewed.

## 1. Introduction

Bionanoscience is a combination of biology and nanotechnology that is used to develop biomaterials, devices, and methodologies at the nanoscale. According to its tremendous applications, nanobiotechnology has become a rapidly developing area of science research [1]. Nanosystems can be fabricated by micromachining to create ever-smaller features (top-down) and/or to incorporate modified smaller features into the macromaterial (bottom-up) using assembly and/or supramolecular chemistry techniques. Biomaterials such as viruses, DNA, proteins, and RNA that exhibit an amazing diversity of highly superior structures are suitable nanostructures for the creation of new material [2,3,4,5]. Viruses have great deal of interest in the field of nanotechnology due to their small size, structural symmetry, ease of functionalization, monodispersity, and ability to self-assemble. Taking advantages of their prescribed shape together with the physiochemical functionality, viruses offer endless benefits over organic and inorganic chemistry.

Viruses are protein cages that are used for the production of novel nanomaterials in a very precise and controlled fashion. Viruses have a variety of distinct shapes, most commonly icosahedrons and rod-shaped. Genetic and chemical modification enables the insertion or replacement of selected amino acids on virus capsids for uses ranging from bioconjugation to mineralization.

**Figure 1:**
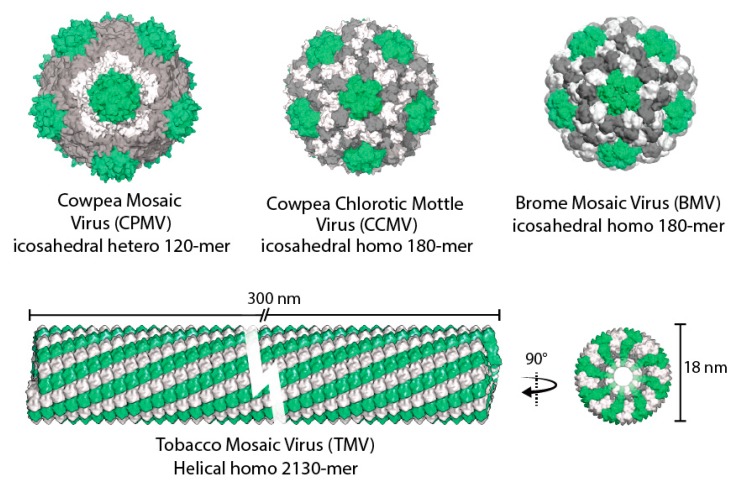
Structural comparison of some plant viruses used to build nanoscale materials. The colors are added to highlight the morphological units and are not necessarily indicative of different protein sequences. All the structures are generated using UCSF Chimera.

**Figure 2:**
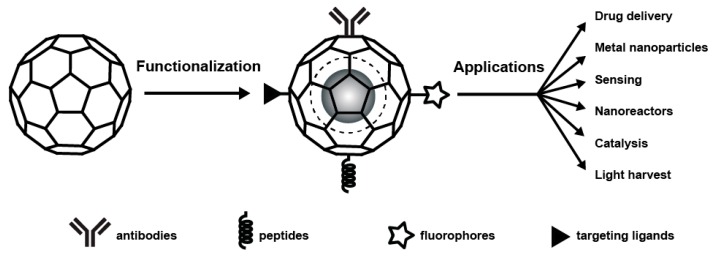
Schematic representation of plant virus functionalization and applications.

In recent years there has been increased interest in the development of plant viruses as biotemplates for beneficial uses. The applications have been diverse, ranging from biomedicine to sensors. Characteristics that make many plant viruses attractive for these studies are their relative simplicity, including ease of purification, the lack of membranes, and simple one or two protein capsid assemblies that are structurally defined. In addition, as plant viruses are phytophages that utilize plants as hosts, they are mostly non-infectious to animals and humans. Furthermore, plant viruses often have wide range of stabilities to temperature, pH, salt, chemicals, and protease degradation. They also differ in terms of structural plasticity assembly and disassembly parameters and electrostatic interactions. The icosahedral (sphere-like) viruses include cowpea chlorotic mottle virus (CCMV), cowpea mosaic virus (CPMV), brome mosaic virus (BMV), and the rod-shaped tobacco mosaic virus (TMV) have been investigated as ideal platform in variety of applications in nanotechnology (Figure 1). Three distinct surfaces in viruses can be explored: the interior, the exterior, and the interface between subunits. Through genetic or chemical modification, each of the surfaces has potential plasticity for the design of novel functional nanomaterials (Figure 2).

## 2. Plant Viruses as Biotemplates

### 2.1. Cowpea Chlorotic Mottle Virus

The cowpea chlorotic mottle virus (CCMV) belongs to the Bromovirus group of the Bromoviridae family [6]. CCMV capsids are approximately 28 nm in diameter, form an icosahedral shell, which defines a 18 nm diameter interior cavity (T = 3 symmetry) [6]. The quaternary structure of the CCMV displays 32 prominent capsomers: 12 pentamers and 20 hexamers. CCMV capsids are assembled from 180 identical coat protein monomers to form hollow structures that look like closed spherical shells [7]. It was first reported by Trevor Douglas and Mark Young in 1998 as a constrained reaction vessel for nanomaterials synthesis to template the mineralization of inorganic materials that contain polymer chains [8].

The most profound property of the CCMV is that the viral capsid can undergo a pH and metal ion-dependent structural transition called gating, which increases its porosity and allows materials to enter. At pH > 6.5, and in the absence of divalent metal ions, the viral capsid undergoes a reversible swelling (an approximate 10% increase in diameter), allowing the ions to freely diffuse into the interior cavity. The swelling capsid has open pores of 2 nm in diameter with 60 separate permitting ions to dispread through the interior and exterior [6]. When the pH is < 5.0, two effects are induced: (I) a pH dependent oligomerization of the inorganic species to form large polyoxometalate species, which are readily crystallized as ammonium salts, and (II) the structural transition of the CCMV cage from the swollen form to the nonswollen form, thus trapping the inorganic material within the viral cage [8,9].

### 2.2. Cowpea Mosaic Virus

The cowpea mosaic virus (CPMV) is a member of the Comovirus group of plant viruses. It can also be used to strengthen the growth of inorganic materials [9]. Its structure has been identified to a 2.8 Å resolution [10]. CPMV capsids have a diameter of approximately 28 nm and a pseudo-T3 icosahedral symmetry made up of 60 copies of large (L) and small (S) coat proteins and two ss-RNAs contained in the cavity of the capsid protein assembly [11]. Of these, trimers at the threefold axis, made up by 60 large coat proteins (L, domains B and C), fold into two jelly roll β-sandwich domains, and 12 pentamers at the fivefold axis, made up by small coat proteins (S, domain A), fold into one jelly roll β-sandwich domain. Asymmetric units are formed by these three domains. The CPMV is capable of exterior display and encapsidation of molecules which makes the cowpea mosaic virus a powerful noninvasive imaging tool and an appropriate delivery system for therapeutics due to its icosahedral protein coat shape. The CPMV is considered as one of the best investigated viruses and has been widely used in bionanotechnology [11,12].

### 2.3. Brome Mosaic Virus

The brome mosaic virus (BMV) was first extracted from bromegrass in 1942 [13]. Its genomic organization has been known since the 1970s, and it has been fully sequenced with clones on a commercial basis since the 1980s [14]. The BMV is a small, positive-stranded, icosahedral RNA plant virus in the alphavirus-like superfamily. The BMV is a member of the genus Bromovirus, family Bromoviridae. BMV capsids have also been used to encapsulate nanoparticles and have a spherical structure with icosahedral symmetry and an inner diameter of approximately 18 nm. The capsid is comprised by 180 identical copies of one coat protein arranged in T = 3 symmetry. Particles with sizes up to 16 nm can also be incorporated, and this size is close to the inner diameter of the BMV capsids.

The BMV has a special genome that is classified into three kinds of 5′-capped RNAs [15]. RNA1 (3.2 kb) contains a protein called 1a (109 kDa) including both an N-proximal methyltransferase domain and a C-proximal helicase-like domain. RNA2 (2.9 kb) contains the 2a protein (94 kDa): the RNA-dependent RNA polymerase responsible for the replication of the viral genome [16]. RNA3 (2.1 kb) contains two proteins, the 3a proteins (involved in cell-to-cell migration during infection) and the coat protein (for RNA encapsidation and vascular spread). The 3a and coat protein are indispensable for the infection of the system in plants rather than RNA replication [17,18]. Similar to the CCMV, the BMV has a pH and an ion dependent swelling mechanism [19].

### 2.4. Tobacco Mosaic Virus

The tobacco mosaic virus (TMV) is a helical plant virus consisting of 2130 identical subunits arranged helically around an RNA genome in its native form. The TMV is 300 nm in length and 18 nm in diameter [20]. It is a rod-shaped plant virus with 4 nm cylindrical central pore. The internal and external surface of the capsid protein consists of repeated patterns of charged amino acids such as lysine, arginine, glutamate, and aspartate. Due to its morphology, it is convenient to create nanowire-like materials using the TMV rod [21,22]. The TMV exhibits excellent stability at pH values of 3.0 to 9.0, temperatures up to 90 °C, and is resilient to several polar organic solvents such as methanol, ethanol, acetone, DMSO, and tetrahydrofuran [23]. This makes the TMV an ideal inorganic template for nanofabrication to grow metal or metal oxide nanoparticles such as iron oxides, iron oxyhydroxides, cadmium sulfide, lead sulfide, gold, nickel, cobalt, silver, copper, CoPt, FePt, and silica [24,25,26,27,28].

## 3. Chemical Modification and Genetic Engineering of Plant Viruses

Viruses offer three different surfaces that can be exploited: the exterior, the interior, and the interface between the subunits. These interfaces can be used to conduct chemical and genetic modifications for a variety of purposes. The external capsid surfaces possess a large number of selectively addressable amino acids allowing decoration with a large number of molecules. The functional groups on the capsid surface, amine groups from lysine, and carboxylate groups from aspartic and glutamic acids offer precise sites for chemical modification using various techniques such as covalent coupling [29,30,31], click chemistry, and the copper (I)-catalyzed azide-alkyne [3 + 2] cycloaddition reaction [32]. As the thiol-derived cysteine side chain is the most popular group for conjugation, various viruses are subjected to site directed mutagenesis to cysteine [33,34,35,36]. Insertion of unnatural amino acids is also feasible, allowing for more diverse chemical modifications [37,38].

Small molecules have access to the viral inner cavity with imperceptibility to large molecules. Based on this property, it is possible to allow the internal cavity of virus-like particles (VLPs) to be used as a nanotemplate and nanoreactor. In addition, the virus exterior surface is a powerful platform not only for chemical modification and genetic modification, but also for multivalent ligand display. A key component of assembly is the virus architecture interface, which provides additional ways to manipulate the capsid architecture.

A series of amino acid side chains (e.g., lysine, cysteine, tyrosine, and histidine) are located on the protein shell of the viral particles and these amino acid side chains are applicable to fluorescent and medical imaging dyes, proteins, or small molecule therapeutics and sensors or cell-penetrating peptides of the desired reagents for chemical-conjugated reactive sites. Targeting the conjugation site at a particular location on the inner or outer surface can be accomplished by genetic engineering of new residues. For example, through this method, a three dimensional (3D) array of Au nanoparticles (1.4–5 nm) can be produced on the CPMV surface [39,40]. The addition of unique cysteine residues on the surface of the CPMV resulted in the successful attachment of Au nanoparticles to defined spatial positions [41]. CPMV scaffolded Au particles are then interconnected via thiol conjugations to produce conductive nanowires and blocks. It is a novel method to produce nanoscale electrical circuits by using the uniform and repeating patterns of plant virus nanoparticle (PVN) reactive sites to design and control the contacts between 3D arrayed PVNs.

The CCMV has a positive inner surface, which hinders iron nanoparticle synthesis. However, protein engineering can change the charge of the inner surface through mutagenesis. After that, iron compound nanoparticle synthesis is possible inside the CCMV [42]. In conclusion, proteins can be good templates for nanoparticle synthesis and have the advantage of yielding uniformly sized nanoparticles. Protein engineering can expand the application of the biotemplates. Discovering more biotemplates and modifying the functions of proteins are the focus of future endeavors in this area.

## 4. PVN Assembly 

Accurately patterning and integrating biotemplates into devices to take advantage of the nanosize and enhanced functionality is a great challenge in nanoscience applications. Here are some methods to enhance the applications of several functionalized PVN surfaces.

### 4.1. Two-Dimensional Surface Assemblies

Using evaporation methods with drop-and-dry or solvent impregnation, the surface alignments of the icosahedral and filamentous plant-based PVNs have been accomplished. The PVN concentration and the intensity of the surface attractive force affect the formation of the PVN film [43,44]. The effect of PVN-coated surfaces on cellular responses and differentiation is based on their homogeneity and multivalent properties [45,46]. The coated surfaces of the TMV particles play a facilitating role in the differentiation of mesenchymal stem cells into bone producing osteogenic cells. Additionally, this effect is also present for 24 h where the stem cells exhibit significant bone morphogenetic protein-2 (BMP-2) induction and coalescence to form bone-like nodules [47]. In a subsequent study, surface-specific TMV PVNs chemically cross-linked with RGD adhesion peptides that also enhanced differentiation into osteoblasts [48]. Based on these studies, we understand that plant-based PVNs providing multivalent display scaffolds can exhibit nanotopographical features that promote cell adhesion and differentiation. Advincula et al. reported the formation of hierarchical CPMV nanoparticle assemblies on colloidal-patterned, conducting polymer arrays [49] where the electrostatic interaction between the CPMV and the pyrrole-based copolymers drove the site-specific assembly on the nanopatterned conduction polymer array.

Surface displayed metal nanoparticles exhibit unique material properties and an increased surface area of the nanoscale catalysts present enhanced activities. An important way to control the catalytic reactions is reflected by the ability to adjust the position, size, and spatial density of the catalyst. PVN provides a unique backbone for the production, anchoring, and display of key nanocatalytic materials. For instance, Yang et al. studied the production and display of Pd nanoparticles of a defined size with a diameter of 5–15 nm; their distribution can be controlled by TMV PVN [35]. The TMV templated Pd nanocatalysts in the catalysis reactions of the toxic environment pollutant hexavalent chromium have a higher catalytic activity per unit Pd mass than commercial Pd/C catalysts [50].

### 4.2. 3D Assemblies

**Figure 3:**
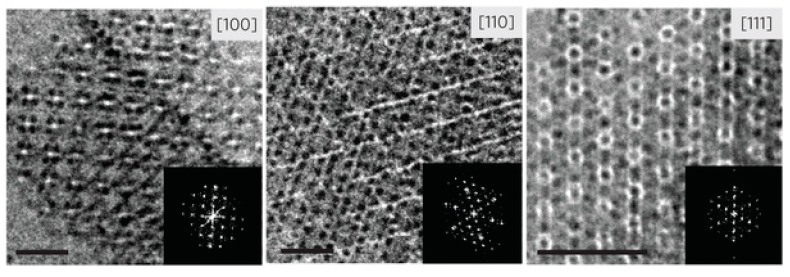
Cryo-TEM images of superlattices of CCMV with encapsulated gold nanoparticles viewed along [100], [110], and [111] zone axes (scale bar 50 nm, inset is image Fourier transform). Reprinted from a past study [56]. Copyright 2012 Nature Publishing Group.

**Figure 4:**
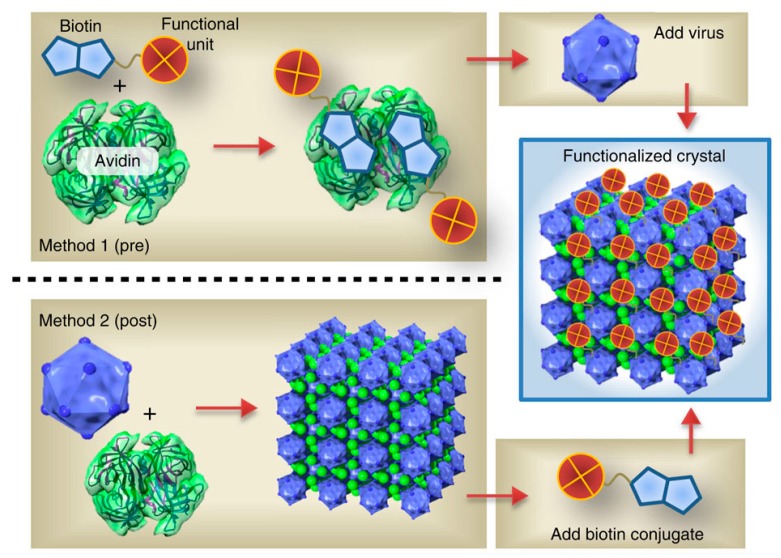
Pre- and postfunctionalization approaches of CCMV-avidin crystals. Electrostatically assembled CCMV (acidic)/avidin (basic) co-crystals can be functionalized selectively with biotin-tagged functional units. Method 1: Prefunctionalization of avidin with the biotinylated functional unit followed by addition of the virus particles. Method 2: Assembly of the crystals followed by postfunctionalization with the biotinylated agent. Copyright 2014 Nature Publishing Group.

The icosahedral viruses are highly consistent 3D building blocks suitable for multidimensional nano- and mesoscale assembly [51,52]. For instance, through biotin-streptavidin crosslinking, CPMV and CCMV can form layered 3D surfaces structures [53,54]. Depending on the functional uniqueness of the layer-by-layer assembly of PVNs, micron ordered particle assemblies can be produced by this method. Cornelissen and coworkers utilized optically sensitive dendrimers to assemble the CCMV virus-like nanoparticles in a highly ordered fashion without modifying the cages [55]. Optical triggering induced the controlled decomposition and charge switching of dendrons, which resulted in the loss of multivalent interactions and the release of virus particles. Kostiainen et al. produced 3D superlattices using a tunable electrostatic attraction between negatively charged CCMV particles and positively charged gold nanoparticles [56] (Figure 3). They further explored this approach and demonstrated that superlattices with, not only face-centered-cubic (FCC) structures, but also hexagonal-close-packed (HCP) structures and body-centered-cubic (BCC) structures, by changing the type and size of the mediator [57] (Figure 4). The strategies including both gold nanoparticle and dendrimer-mediated assembly suggest that size matching and charge anisotropy could be applied to direct highly ordered assemblies in a range of virus-like particle systems. The filamentous plant virus, which represents a high aspect of structure, has the potential to significantly enhance the surface area if patterned in a vertical manner. To address this potential and to promote vertical alignment and surface attachment, rigid rods of TMV were genetically engineered to achieve the goals [58].

Using the known 3D structure of TMV, a cysteine codon was inserted at the N-terminus of the CP ORF to create a new mutant TMV1cys [59]. Due to the presence of thiol-metal or thiol-charge interactions, the localization of the 1cys mutation assists in the attachment and vertical positioning of the virus rods on various surfaces including gold, stainless steel, SU-8 polymer, and Teflon™ [58,60,61]. Despite the mutation of the *N*-terminal 1cys, which is recessed in the groove and partially covered by the C-terminal arm of the CP, this site may inhibit direct contact between the cysteine-derived thiol groups and the metal surface, except at the 3′ end of the virion rod where the thiol group is sufficiently exposed to make direct surface contact. ELD mineralization of the subsequent surface-assembled TMV1cys yielded a uniformly coated virus template comprising a fused metal coating with a thickness of 15 nm. In addition, the virus stick vertically assembled to the surface, so the available area significantly increased by an order of magnitude [58].

**Figure 5:**
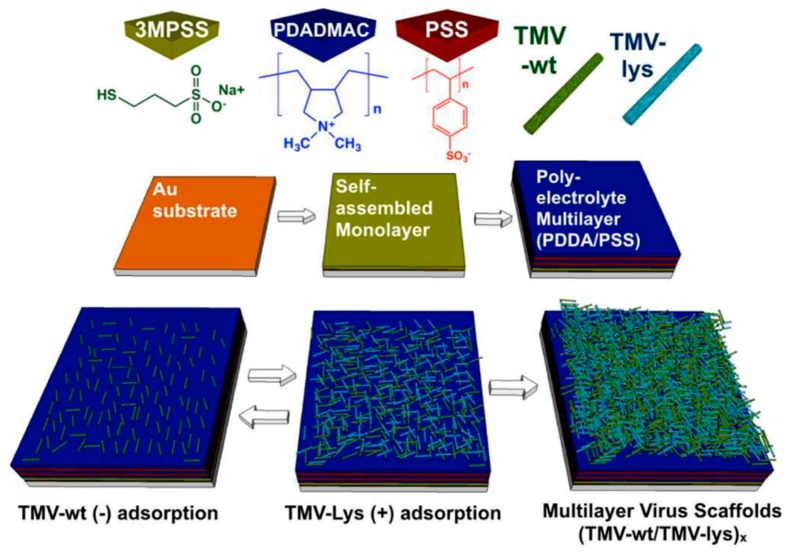
Sequential formation of multilayer virus scaffolds via layer-by-layer deposition of tobacco mosaic virus (TMV-wt) nanoparticles and mutant TMV particles with lysine residues (TMV-lys). Copyright 2016 Royal Society of Chemistry.

The bottom-up assembly of nanoscale building blocks provides unique solutions in achieving more complex and smaller morphologies with a wide-range of applications. Charged TMV (TMV-wt and TMV-lys) nanoparticles were employed in constructing multilayered fibrous networks via electrostatic layer-by-layer deposition (Figure 5) [62]. The electrostatic interaction between TMV-wt and TMV-lys nanoparticles was the driving force in building the multilayer TMV constructs. The multilayer TMV scaffolds had the capability to support the proliferation and differentiation of osteogenic cells and increase in cell attachment with increasing layer deposition.

Overcoming the problems associated with the abnormal metallization of nontemplated particles can be represented by surface-assembled virus particles prior to the inorganic coating [63]. Attachment of the viral template to the surface allows these nontemplated clusters to be washed away. This process provides a simple and powerful way to increase areas for nanosurfaces and integrate directly into the device interface. The potential of these nanotemplates to be integrated into traditional manufacturing processes is reflected in the microfabrication approach without disrupting their structure or activity.

## 5. Applications of Plant Viruses Based Nanomaterials 

### 5.1. Biomedical Applications

Virus-based nanoparticles (VNPs) have received great attention in recent years; plant viruses have been used in materials science research and have the potential for application in nanomedicine [4,64]. As viruses possess the property of self-assembling around the cargo (the genome) and delivering this cargo to specific cells and tissues, they are ideal candidates for specific delivery sites for therapeutic or contrast agents. In fact, several VNP technologies for clinical trials of gene delivery and oncolytic virus therapy are being developed and applied. Due to their high degree of symmetry, multivalency, monodispersity, and genetic or chemical programmability, VNPs have become promising materials. Most VNP structures have been resolved to allow a high degree of spatial control of the tailored atomic resolution. VNPs can be modified by imaging contrast agents, therapeutic moieties, or targeting ligands (e.g., peptides or antibodies) in a chemical selective bioconjugation reaction. 

#### 5.1.1. Magnetic Resonance Imaging

Magnetic resonance imaging (MRI) is a technique that visualizes the structure and metabolites within the capsid and therefore has the potential to detect physiological processes in vivo. Among them, the contrast agent and macromolecular platform can further enhance imaging sensitivity. Paramagnetic gadolinium (Gd^3+^) is frequently applied as a MRI contrast agent, however, it has to be used in a chelated form to reduce the toxicity, usually with DOTA tetraazacyclododecane tetraacetic acid (DOTA) or diethylenetriamine pentaacetic acid (DTPA) [65]. After conjugation with such nanoparticles, a decrease in the molecular tumbling rate of gadolinium ions results in an increase in the longitudinal relaxation rate [66]. Finn and Manchester group modified azide-displaying viruses with an alkyne-modified DOTA ligand [67] that showed an increased *T*_1_ relaxivity relative to free Gd (DOTA) complexes after loading with gadolinium. These reagents, determined as nontoxic in vivo, were used for the MRI imaging of live mice [68]. The increase in local concentration can be caused by the multivalent display, and both of these events can increase the sensitivity. Since Gd^3+^ or other lanthanides can increase the relaxation rate, differences in MRI signals can be detected regardless of where they are present, and the contrast level by many factors can be finally obtained. A multifunctional MRI contrast and photodynamic therapy agent (chelated Gd^3+^ and Zn^2+^ phthalocyanine dye) has been incorporated inside capsids of the CCMV protein which represent the first step towards the consecution of fully self-assembled protein cage nanoparticles for multimodal imaging and therapy [69].

The covalent attachment of chelated gadolinium ions to the TMV leads to the enhanced ionic relaxivity of the Gd ions based on reduced tumbling rates. Multivalent display leads to a relaxivity per nanoparticle that is four times higher than other VNP contrast agents [70]. Steinmetz et al. developed a high contrast-to-noise ratio, molecularly targeted MRI contrast agent using TMV nanoparticles [71,72]. To achieve dual optical and MRI, the TMV was modified to carry near-infrared dyes and chelated Gd ions. The probe targeted atherosclerotic plaques. On the basis of the multivalency and multifunctionality, the TMV-based MR probe showed an enhanced detection limit, 400 times lower than the typical clinical dose [73].

#### 5.1.2. Therapeutic Delivery System

The application of plant viruses to drug delivery is still at an advanced stage of development. However, the plant virus can be devised with new properties based on the unique requirements of the targeted drug delivery. After chemical functionalization of the surface of plant virus particle, they have the property of stealth, thereby extending their circulatory half-life or targeting ability in the body to help deliver the drug to the diseased tissue. The amino acid sequence of the viral coat protein is genetically manipulated to produce a protein cage of a particular chemical structure and the conformation of the protein cage in the external environment can be directed to disassemble and then be reassembled in vitro to exchange the genomic material of the native viral foreign cargo [74].

Without further replication, plant viruses enter mammalian cells, which make them suitable as therapeutic delivery systems. For implementation in nanomedicine, the therapeutic cargo is either loaded into the inner cavity or conjugated to the interior or exterior surfaces of the capsid. Viral nanoparticle surfaces can be chemically or genetically modified to target particular cells including cancer cells and specific cells of the immune system. Currently, virus-based nanoparticles have been widely used in vaccine platforms [75,76,77,78] and biologically active molecules including genes [79,80], proteins [81], and drugs [82,83,84,85,86,87,88]. For gene therapy, a prominent example is the development of CCMV to deliver mRNA cargos. A large range of lengths and sequences of single-stranded RNA molecules were delivered to cells using CCMV VLPs with lipofectamine complexes and were released in the cytoplasm of mammalian cells, leading to the expression of the reporter-enhanced yellow fluorescent protein. The CCMV capsid protects the packaged RNA against degradation during storage and delivery [80]. Targeting can be achieved by chemically or genetically adding ligands which bind to receptors overexpressed on cancer cells. For example, VNPs have been targeted using RGD peptides [89], epidermal growth factor (EGF) [90], and folic acid [84,91,92]. In addition, to increase the plasma half-life and produce the immunogenicity of therapeutic agents, the chemical conjugation of PEG has often been used [34,85,93,94,95,96].

VNPs have been used as nanocarriers for a variety of clinically approved chemotherapeutics. Complexation of the therapeutic compound with VNPs possesses several advantages, including an improvement in pharmacokinetics and enhanced drug delivery to disease sites while overcoming off-target effects [4]. Doxorubicin has been used to treat a variety of cancer types including breast, ovarian, and prostate, among others. However, high off-target toxicity limits the administered dose, reducing treatment efficacy [97]. High aspect ratio TMV nanotubes as well as TMV-derived disk-shaped nanoparticles have been conjugated with doxorubicin through covalent attachment [98,99]. Spherical TMV nanoparticles have also been infused with doxorubicin by mixing doxorubicin and TMV during the heat-transition from rod-to-sphere. Either method yielded doxorubicin-loaded TMV formulations that exhibited potent cytotoxicity against breast cancer cells [98].

**Figure 6:**
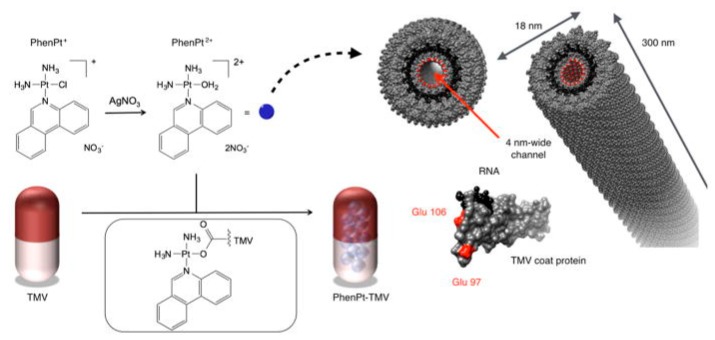
Schematic for loading of phenanthriplatin into TMV. Reprinted from a past paper [102]. Copyright 2016 American Chemical Society.

Noncovalent methods allow for higher loading efficiency as more cargo molecules can be loaded in the entire cavity when compared to the covalent attachment, which uses the internal exposed side chains to conjugate the cargos [84]. For example, approximately 900 doxorubicin molecules can be encapsulated into the internal cavity of the hibiscus chlorotic ringspot virus (HCRSV) [100], whereas only 300 doxorubicin molecules can be conjugated to the internal surface of the cowpea mosaic virus (CPMV) [83]. Although most protein-based nanocarriers are spherical in morphology, the Francis group developed nonspherical nanomaterials based on a TMV mutant that displayed a highly stable double-disk state. The TMV mutants were modified with both doxorubicin and PEG5000 through orthogonal bioconjugation strategies and were successfully used to deliver the drug to glioblastoma cells in vitro [88]. Recently, Steinmetz and coworkers demonstrated the TMV-assisted delivery of the drug candidate phenanthriplatin, which is a cisplatin derivative up to 40× more potent than contemporary platinum therapeutics [101,102]. They established a noncovalent drug loading method yielding therapeutic TMV carrying approximately 2000 phenanthriplatin moieties in its central channel. The association of phenanthriplatin and TMV is electrostatically driven (the dication of phenanthriplatin interacts with the negatively-charged interior of TMV). The phenanthriplatin-loaded TMV exhibited matched efficacy in a cancer cell panel when compared to free phenanthriplatin. In vivo tumor delivery and efficacy was confirmed using a mouse model of triple negative breast cancer [102] (Figure 6).

Environmentally-triggered structural changes of nanocages, for instance CCMV, allow the entry of the drug into their hollow interiors by changes in pH or ionic strength. The pores are open at neutral pH in the presence of CaCl_2_, which enables mixing of the drug and the medium; at lowered pH, the pores close and thereby capture the drug. Using this property, phthalocyanine (ZnPc), a light-absorbing molecule, was encapsulated into CCMV capsids. The ZnPc-containing CCMV capsids were eaten by RAW 264.7 macrophage cells that led to their deaths, indicating the applicability of CCMV-ZnPc for photodynamic therapy (PDT) [103]. PDT is a form of phototherapy involving light and a photosensitizing chemical substance, and is used in conjunction with molecular oxygen to elicit cell death. Targeted delivery of PDT by VNPs allows for increased clinical applications through the reduction of the host-cell/nontarget tissue damage [104,105]. *Staphylococcus aureus*, a pathogenic bacterium, was inactivated through treatment with dual-functionalized CCMV with a photosensitizer and a targeting ligand [106].

### 5.2. Enzymatic Nanoreactors and Catalysts

Enzymes can also be loaded into the virus capsid to create nanoreactors. In nature, enzymes are found in the limited space of cells. Encapsulation of the enzyme within the viral capsid provides the possibility of studying enzymatic behavior in a cell-mimicking environment. In artificial nanoreactors, the envelopment of enzymes as a biomimetic system are expected to help us understand the interaction of enzymes in active and confined spaces. The capsid of the cowpea chlorotic mottle virus (CCMV), a man-made nanoreactor, can be used to encapsulate a variety of proteins within it. By controlling the encapsulation process, it is able to load the exact amount of protein (*Pseudozyma antarctica* lipase B and EGFP) in the CCMV capsid to study the activity of this structure. In the case of enzymes, studies have shown that in the encapsulation process, the apparent increase in the overall rate of reaction is almost uninfluenced by the amount of enzyme in the capsid. This result is due to the very high limiting molarity of the enzyme in the capsid, leading to very rapid formation of the enzyme-substrate complex, and highlights the importance of a small volume for efficient multi-enzyme cascade catalysis [107]. Based on the monodisperse nature of the viral capsid, it is primarily used to study the controlled synthesis of biomineralization and inorganic nanomaterials. Recently, there has been increasing interest in the use of viral capsids in a larger space to incorporate enzymes or proteins to mimic a restricted cellular environment.

In order to prevent the holder body from diffusing, the use of the catalyst in the protein cage needs to be included in the category and minimum size hole through the housing. Ueno and his colleagues prepared a selective hydrogenation biocatalyst by the in situ chemical reduction of palladium (II) ions in the apoferritin cavity [108]. In this case, the 3-fold channel of the protein shell allowed [PdCl4]^2−^ to pass through, and the protein coating diffused into the apoferritin cavity with the resulting palladium nanoclusters not being able to leave the protein cage. After encapsulation, the catalytic material could be distinguished as the size of the olefin to be hydrogenated.

**Figure 7:**
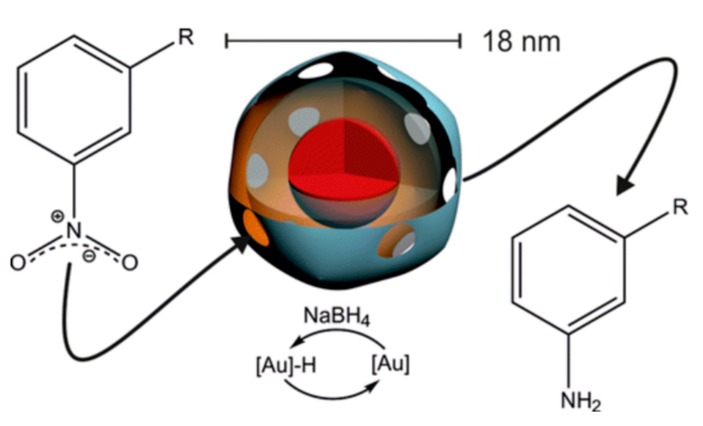
Colloidal gold nanoparticles were encapsulated in cowpea chlorotic mottle virus cages which catalyzed the reduction of nitroarenes with different substituents. Copyright 2016 American Chemical Society.

Encapsulation of enzymes or synthetic polymers is different and because of their larger size; they struggle to diffuse through the holes of the protein cage. However, some RNA plant viruses can be reversibly disassembled/assembled by changing the medium pH and salt concentration in order to provide a simple mechanism to bind large species. The most widely studied virus is the cowpea chlorotic mottle virus (CCMV) [7] with an outer diameter of 28 nm and an inner diameter of 18 nm. When the pH of the medium is above 7.5, the CCMV virus particles break-down into protein dimers and RNA. After removal of the RNA, the purified viral CP subunit can self-organize and empty the capsid if the pH is reduced to 5.0. pH-dependent behavior has been used as a method for encapsulating proteins [109], inorganic nanoparticles [8], and anionic polymers for these species. The incorporation of a single horseradish peroxidase (HRP) enzyme molecule into the inner cavity of CCMV has been reported using the capsid disassembly/assembly properties [109]. Under the experimental conditions used, no more than one HRP molecule per virus particle was encapsulated. Cornelissen et al. successfully constructed nanoreactors based on the CCMV protein cage and a gold nanoparticle, which could catalyze the reduction of nitroarenes (Figure 7) [110]. The electronic effects and reduction rate constants are reported to have originated from the limited pore sizes and charged exterior/interior of the cage. The catalytic nanoparticles can be reused after isolation and re-encapsulation in the protein cage.

Recently, mineral Co_3_O_4_ has been synthesized in rod-like tobacco mosaic viruses by Ludwigs and coworkers, which demonstrated superior catalytic performance in the oxygen evolution reaction when compared to a commercial nanopowder [111].

### 5.3. Light-Harvesting System Based on the TMV

**Figure 8:**
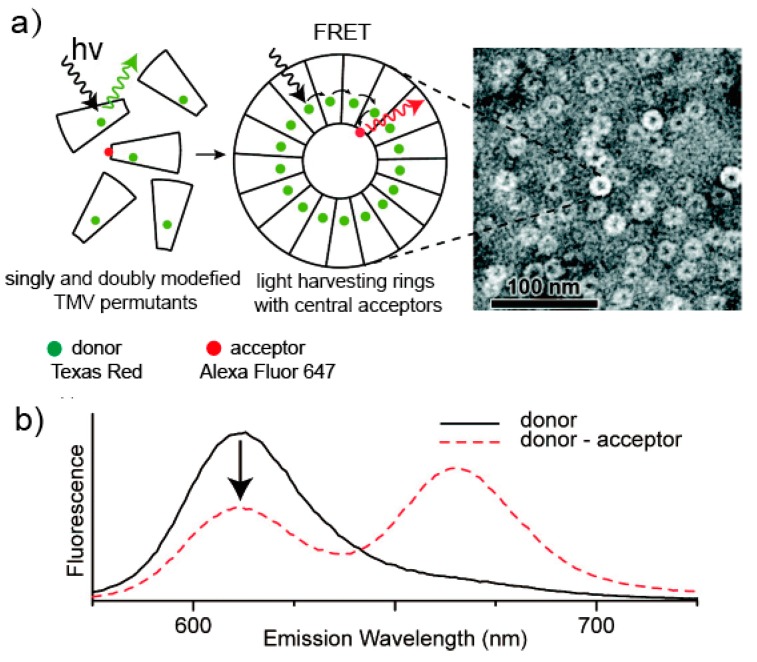
Site-specific labeling of cpTMVP with donor (Texas Red) and acceptor (Alexa Fluor 647) chromophores to measure assembly. (**a**) A cartoon representation of a cpTMVP disk shows the approximate locations of donor and acceptor chromophores. Double modification does not perturb the structure of disks as measured by TEM. (**b**) Overlay of the emission spectra of donor only and donor-acceptor systems shows the assembly-dependent FRET. Reprinted from a previous paper [116]. Copyright 2010 American Chemical Society.

Viral capsids provide a means to precisely position chromophores with spatial control at the sub-nanometer level. The tobacco mosaic virus coat protein can serve as a convenient template for the synthesis of chromophores into light-harvesting arrays [112,113,114]. An interesting aspect of this virus is that when the ionic strength and pH of a solution changes [20], the monomers can self-assemble into different nanoscale structures and can form a double-layer disk composition comprising of 17 monomers per layer or an extended helical rod comprised of 16.33 monomers per turn. The disk and rod structure can provide a rigid frame for establishing and maintaining an accurate distance relationship between several microns of monomer (for rods), which is a very challenging technique for protein without capsid synthesis methods. TMV coat proteins were covalently labeled with either a donor or an acceptor from a Förster resonance energy transfer (FRET) pair and then assembled into disk or rod structures at a controlled ratio of donors to acceptors. During the assembly process, it is possible to adjust the TMV capsid by simply adjusting the ratio of monomers tagged for each component [112,115]. For instance, the TMV virus was covalently modified at both the *N*-terminus and at an introduced cysteine residue with two different chromophores that undergo FRET to produce a “light harvesting rod” [116] (Figure 8).

Although TMV is the first structurally characterized virus, research on the preparation of new materials for covalent modification has not been reported since 2005. In the first report on the subject, Francis described the use of diazotization chemistry to link tyrosine residues on the outer surface of a native capsid with carbodiimide chemistry to link an amine to an internal channel surface. According to this method, many different chromophores, ion chelators, and polymer chains have been added to the viral capsid surface [117]. Through the use of cysteine modification chemistry, porphyrins have been attached to individual capsid monomers to yield conjugates that self-assemble into efficient light harvesting systems containing thousands of interacting groups [118].

### 5.4. Sensor Applications of Biomimetic Nanostructures

The development of nanotechnology has greatly improved the application of biomolecule nanostructures in biosensors and biomedical engineering [119,120,121]. Multifunctional nanoparticles and other nanoscale building blocks have been incorporated into biological molecules and biomolecular supra-structures due to their application in the field of biosensors, biological diagnosis, and treatment. Biomolecule-based nanostructures prepared using biosensors and bioassays have better selectivity and sensitivity than traditional molecular probe-based sensing techniques [122].

With the development of sensing technology in various fields, colorimetric sensors have attracted public attention. With this technique, it is easy to read the results with an acceptable resolution with the naked eye. To a certain extent, these simple colorimetric sensors may even eliminate the need for instruments. Researched by Mirkin and coworkers [123,124,125], the most widely used colorimetric sensor is a scanometric method based on metal nanoparticles. In these cases, the gold nanoparticles are first modified with probe DNA or aptamer and the addition of analytes to the nanoparticle system can be based on DNA hybridization or aptamer-based molecule recognition to promote the aggregation of the gold nanoparticles such that the nanoparticle solution of the color changes significantly. The combination of DNAzyme and gold nanoparticles can be used for the colorimetric sensing of metal ions. For example, in the study of Lu et al., they first reported a colorimetric sensor using DNAzyme-directed gold nanoparticle assembly to produce Pb^2+^, Hg^2+^, adenosine, and cocaine [126,127,128,129]. DNAzyme directly assembly produces DNAzyme-gold NP sensors that are highly sensitive with selectivity for analytical substances. Protein-mimetic metal nanoparticles can also be used for colorimetric sensing [130,131]. According to Li et al., it has been reported that bovine serum albumin (BSA) can be used as a nucleation template, and the average diameter of the biomimetic platinum nanoparticles was 2 nm, which is a typical example [131].

The ability to display selective peptides from the surfaces of VLPs provides a unique opportunity to integrate scaffolded binding peptides into sensor systems for the detection of a range of biological and inorganic targets. TMV VLPs have been successfully demonstrated as receptors in impedimetric and electrochemical sensors for TNT detection. Zang et al. utilized the TMV coat protein to display trinitrotoluene (TNT) binding peptides which selectively bind TNT in solution and thus the diffusion onto a sensing electrode reduced. The measured TNT electrochemical reduction peaks were shown to correspond to the concentration of TNT [132]. This sensing method enables rapid label-free detection of TNT and can be expanded to a variety of electro-active species. Most recently, a capillary microfluidics-integrated sensor system was developed for the rapid assembly of bio-nanoreceptor interfaces on-chip and label-free biosensing [133]. Displaying thousands of copies of the identical receptor peptides FLAG-tags, TMV virus-like particles were used as nanoreceptors for antibody sensing. By using the capillary effect and surface evaporation from an open-channel capillary microfluidic system, the controllable and accelerated assembly of VLP receptor layer was realized on an impedance sensor. Only 5 μL VLP samples formed a dense localized receptor monolayer on the impedance sensor within six minutes at room temperature. This work highlighted the great potential of integrated microsystems in fast and controllable sensor transducers. TMV has also been reported in terms of its use as an advantageous carrier for sensor enzymes and volatile organic compounds [122,134]. In the enzyme-based sensor study, the surface of TMV was equipped with two streptavidin-conjugated enzymes: glucose oxidase (GOx) and horseradish peroxidase (HRP). GOx catalyzes the oxidation of glucose and generates hydrogen peroxide, which can then be reduced to water by HRP in the presence of a substrate. Thus, the two enzymes can be coupled to form a glucose sensing system, where HRP substrate conversion can be used for detection. TMV sticks allowed the immobilization of up to 45-fold higher catalytic activities than control samples with the same input of enzymes [122]. The enzyme-based biosensors could also be created by the immobilization of other enzymes that catalyze other reactions.

CPMV particles have also been used as sensors for the detection of DNA and toxins [135,136,137]. The CPMV viral capsid was applied as a scaffold to increase the fluorescent carbocyanine dye (Cy5) molecules (>40 dye molecules) with controlled intermolecular distances to eliminate self-quenching of the reporter molecules at fixed locations on the viral capsid for the detection of DNA-DNA hybridization with high sensitivity in DNA microarrays [137]. When compared with the most often used detection methods in a microarray-based genotyping assay for *Vibrio cholera* O139, these viral nanoparticles markedly increased assay sensitivity. In a DNA microarray assay, the EF CPMV mutant functionalized with fluorescent dye (Cy5) and NeutrAvidin (NA) was used as a recognition element for hybridized DNA containing biotin. These NA-Cy5-CPMV particles provided a strong signal intensity with low background with a detection sensitivity of 10^1^ genome copies [138]. CPMV has also been used to demonstrate signal enhancement in direct and sandwich immunoassays using antibodies. In sandwich immunoassays for staphylococcal enterotoxin B detection (SEB), the Alexa647-CPMV-anti-SEB complex produces a stronger signal than the mole equivalent of the Alexa647-anti-SEB control demonstrating the advantage of CPMV as a nanoscaffold to couple active biomolecules and a larger number of reporter dye molecules on the same capsid for the detection of toxins. 

## 6. Conclusions

Plant virus-based nanoparticles that are nanocages or nanorods assembled from viral capsid proteins have been widely used as templates to guide the preparation of complex nanostructures. In this review, we described the structural and unique biochemical properties of several plant viruses such as the cowpea chlorotic mottle virus (CCMV), cowpea mosaic virus (CPMV), brome mosaic virus (BMV), and tobacco mosaic virus (TMV). The advantages of plant virus nanoparticles as biological templates in chemical modification and genetic engineering were also analyzed. Nanomaterials developed using viral nanoparticles have superior properties over traditional materials and their potential has been integrated into traditional manufacturing processes that are reflected in microfabrication processes without disrupting their structure or activity. There are many exciting opportunities for potential applications of VNPs or VLPs in biotechnology. Further studies are required to explore the unique self-assembly architectures of more plant viruses to produce new materials for application in the nano biotechnology. In addition, de novo protein engineering and in silico techniques have been rapidly developing in recent years and can play an important role in engineering supermolecular nanomaterials for specific applications.
